# Effects of tannic acid on the immunity and intestinal health of broiler chickens with necrotic enteritis infection

**DOI:** 10.1186/s40104-023-00867-8

**Published:** 2023-05-04

**Authors:** Huiping Xu, Jianyang Fu, Yimeng Luo, Peng Li, Bochen Song, Zengpeng Lv, Yuming Guo

**Affiliations:** 1 Department of Animal Nutrition and Feed Science, State Key Laboratory of Animal Nutrition, College of Animal Science and Technology, China Agricultural University, Beijing, 100193 China; 2 Department of Animal Science, Shandong Agricultural University, Taian, 271018 China

**Keywords:** Broiler chicken, Immunity, Intestinal health, Necrotic enteritis, Tannic acid

## Abstract

**Background:**

In broiler chickens, necrotic enteritis (NE) infection can reduce production performance. Tannic acid has shown great potential as a treatment of NE in broilers. However, the appropriate dosage of tannic acid in NE of broilers and the improvement effect on intestinal health are not very clear. In this study, we aimed to investigate the effects of different doses of tannic acid on the production performance, immunity, and intestinal health of broilers by constructing an NE model with *C. perfringens* infection and determining the appropriate dosage of tannic acid with regard to NE.

**Results:**

Challenged birds showed significant reduction in body weight, villus height, and the ratio of villus height to crypt depth (*P* < 0.05) and increase in the feed consumption gain ratio, intestinal lesion score, and crypt depth (*P* < 0.05). The infection significantly reduced the relative Bacteroidota and *Ligilactobacillus* abundance (*P* < 0.05) and increased the ratio of Firmicutes/Bacteroidota and cecal content of *C. perfringens* (*P* < 0.05). Challenged birds fed diets supplemented with tannic acid showed significantly increased mRNA expression of nutrient transport carriers and intestinal barrier genes and growth performance and reduced serum zonulin and endotoxin levels (*P* < 0.05). Addition of tannic acid to the diet inhibited the inflammatory response by reducing the number of coccidia oocysts in feces and the content of *C. perfringens* in the cecum. Specifically, tannic acid reduced the serum levels of C reactive protein, myeloperoxidase, and specific IgY and ileal mucosal secretory immunoglobulin A levels in the ileal mucosa compared with those in the NE-infected birds. NE-infected birds fed diets supplemented with tannic acid also showed significantly increased relative *Anaerocolumna*, *Thermoanaerobacterium*, and *Thermosinus* abundance (*P* < 0.05); their microbial composition and functional predictions were similar to those of the NC group.

**Conclusions:**

Tannic acid in the diet alleviated NE by enhancing the intestinal barrier and absorption function. The recommended dietary tannic acid additive level is 500–750 mg/kg. Our study findings would be useful in reducing related economic losses in the broiler industry.

## Background

In the poultry industry, the maintenance of healthy intestinal tracts of birds plays a key role in maximizing growth performance. Necrotic enteritis (NE) is a prominent modern enteric disease in broiler chickens caused by *Clostridium perfringens* (*C. perfringens*). This disease causes the destruction of the intestinal mucosal structure and results in the poor digestion and absorption of nutrients and reduced feed efficiency, therefore causing major losses for the industry [1]. In addition, *C. perfringens* can enter the human body through the food chain, threatening public health. Due to the limited use of antibiotic growth promoters (AGP) in the poultry industry, the incidence of NE in chickens has been increasing worldwide. Therefore, effective AGP alternatives are urgently required to prevent and control NE in broiler chickens.

Tannins are water-soluble polyphenols and secondary metabolites in plants [2]. They are classified into two types according to their chemical structures—condensed tannins (CTs) and hydrolytic tannins (HTs). Hydrolytic tannins are phenolic acid polyesters with a *D*-glucose core, such as gallic acid and ellagic acid. The relative molecular weight of HTs is usually 500–3,000 Da, and they are easily hydrolyzed by acids, alkalis, and enzymes. Condensed tannins are composed of flavan-3-ol units connected by carbon–carbon double bonds, also called proanthocyanidins (catechins or epicatechins). Condensed tannins are the most prevalent and typical class of plant tannins, with relative molecular masses ranging from 1,900–28,000 Da and are primarily found in legumes, trees, and shrubs [3].

Tannins have antibacterial, antiparasitic, antiviral, anti-inflammatory, antioxidant, antidiarrheal, and nutrient metabolism-modulating effects [4]. Dietary supplementation of broilers with tannins has been shown to improve growth performance [5, 6] and reduce the abundance of *C. perfringens*, production of toxins such as alpha toxin [7], and the adverse effects of *C. perfringens* and *Eimeria tenella* in chickens [8, 9]. The European Union approved tannins as a novel feed additive for livestock and poultry in 2016. However, it has been shown that the addition of tannins can result in negative effects, including reduction in production performance [10]. The differences in the results may be related to the tannic acid type or dosage used. Therefore, this study was conducted to investigate the modulating effects of different tannic acid additive levels on the intestinal health of broiler chickens co-infected with coccidia and *C. perfringens* to aid the development of more tailored tannic acid application strategies.

## Methods

### Tannic acid

Tannic acid was provided by Hubei Chicheng Technology Development Co., Ltd. (Yichang City, Hubei Province, China). Tannic acid parameters were as follows: tannic acid ≥ 80.0%, moisture ≤ 10.0%, scorch residue ≤ 6.0%, total arsenic ≤ 5 mg/kg, and heavy metal content (as Pb) ≤ 10 mg/kg.

### Experimental design, birds, and diets

The experiment was carried out at the poultry experiment base of China Agricultural University (Zhuozhou City, Hebei Province, China). A total of 630 1-day-old Cobb 500 male broilers were weighed and randomly divided into six treatment groups according to the principle of similar body weight. There were seven replicate cages (1.0 m × 0.7 m × 0.38 m, length × width × height) per treatment and 15 chickens per replicate. They were grouped as follows: (i) negative control group (no tannic acid treatment or NE infection, NC group); (ii) positive control group (NE infection + 0 mg/kg tannic acid, PC group); (iii) PTA1 group (NE infection + 250 mg/kg tannic acid); (iv) PTA2 group (NE infection + 500 mg/kg tannic acid); (v) PTA3 group (NE infection + 750 mg/kg tannic acid); and (vi) PTA4 group (NE infection + 1000 mg/kg tannic acid). Broilers were reared in a room equipped with two-tiered battery cages. All chickens were vaccinated and managed (including light and temperature management) according to routine immunization and management programs of Cobb broilers. Furthermore, all chickens were provided feed and water ad libitum. Diets were formulated according to the Chinese chicken feeding standard (NY/T 33–2004) and were fed in the form of pellets; the nutritional information is summarized in Table 1.

**Table 1:** Ingredients and nutrient content of the chicken feed used during the trial

	**Starter (d 1–14)**	**Grower (d 15–28)**	**Finisher (d 29–35)**
Ingredient, %			
Corn	54.12	58.00	61.79
Soybean meal	32.45	28.00	25.68
Corn gluten meal	5.00	4.00	2.50
Soybean oil	3.00	4.62	4.50
Flour	0.90	0.90	1.05
Calcium hydrogen phosphate	2.00	1.95	1.85
Stone powder	1.00	1.00	1.07
Sodium chloride	0.30	0.30	0.30
*L*-lysine hydrochloride, 78%	0.30	0.30	0.36
*DL*-Methionine, 98%	0.25	0.25	0.19
Threonine	0.10	0.10	0.10
Arginine	0.04	0.04	0.06
Choline chloride, 50%	0.20	0.20	0.20
Broilers—Mineral premix^a^	0.20	0.20	0.20
Broilers—Vitamin premix^b^	0.03	0.03	0.03
Phytase 10,000	0.01	0.01	0.02
Zeolite	0.10	0.10	0.10
Total	100.00	100.00	100.00
Nutrient content^c^			
Metabolic energy, Mcal/kg	2.99	3.10	3.10
Crude protein, %	22.39	20.12	18.5
Lysine, %	1.29	1.17	1.14
Methionine, %	0.61	0.57	0.48
Cystine, %	0.93	0.86	0.75
Threonine, %	0.92	0.83	0.77
Calcium, %	1.08	1.05	1.03
Available phosphorus, %	0.44	0.42	0.40

^a^per kg of trace element premixed feed: copper, 8 g; iron, 40 g; zinc, 55 g; manganese, 60 g; iodine, 750 mg; selenium, 150 mg; cobalt, 250 mg

^b^per kg of vitamin premix feed: vitamin A, 50 million IU; vitamin D_3_, 12 million IU; vitamin E, 100,000 IU; vitamin K_3_, 10 g; vitamin B_1_, 8 g; vitamin B_2_, 32 g; vitamin B_6_, 12 g; vitamin B_12_, 100 mg; niacin, 150 g; *D*-pantothenic acid, 46 g; folic acid, 5 g; biotin, 500 mg

^c^Calculated values based on the experimental diet analysis

### *C. perfringens* challenge

Necrotic enteritis was induced in chickens according to Wu et al. [11] with slight modifications. On d 19, birds in the PC and PTA1–PTA4 groups were orally inoculated with 1 mL of a 25-fold dose of attenuated quadrivalent coccidia vaccine suspension (Foshan Standard Bio-Tech Co., Ltd., Foshan, China). Chickens in the NC group received 1 mL sterile PBS. Chickens were orally gavaged with 1 mL *C. perfringens* type A CVCC52 (3 × 10^8^ CFU/mL) per day on d 22–28, except for broilers in the NC group. *C. perfringens* CVCC52 produces α toxins and does not produce NetB toxins. The birds of NC group received 1 mL sterile fluid medium.

### Growth performance

All broilers were weighed at 19, 28, and 35 days of age in all replicates after 12 h of fasting. Body weight, feed consumption, and the feed consumption to weight gain ratio (F/G) were calculated at different experimental periods. The number of dead chickens per day were recorded and the mortality was calculated.

### Sample collection

One chicken with the average body weight was selected from each replicate at 28 and 35 days of age, and blood was collected from the wing vein, followed by intravenous injection of sodium pentobarbital at a dose of 30 mg/kg body weight. After the anesthesia, the chickens were bled through the jugular vein and sacrificed. The middle region (1 cm) of the ileum was excised and immediately fixed in 4% paraformaldehyde for intestinal morphological examination. Approximately 2 g of chyme from the middle ileum was collected aseptically into sterile tubes; the mucosa of the middle ileum was then gently scraped with a slide, collected in sterile tubes, and stored at −80 °C until further analysis.

### Intestinal NE lesion scoring

At 28 days of age, one chicken in each replicate was anesthetized and sacrificed. The intestinal cavity was opened, and chyme was removed to observe the lesions in the intestinal wall. The scoring rules for intestinal lesions described by Dahiya et al. [12] were adopted; the specific scoring criteria were as follows: 0 = normal intestinal appearance; 0.5 = severe congestion in serous surface and mesentery of the small intestine; 1 = the intestinal wall became thin and brittle, with red silting spots (more than 5); 2 = the intestinal wall appears needle-like necrosis or ulceration, and there is a small amount of gas in the intestinal cavity; 3 = flaky necrosis or ulceration of the intestinal wall, gas-filled intestines and small blood spots, and 1–2 cm long necrotic spots; 4 = diffuse necrosis, significant bleeding, and large intestinal gas.

### Fecal coccidia oocyst counts and determination of intestinal *C. perfringens* concentration

The number of oocysts released after coccidiosis infection in broilers was dynamically monitored for four consecutive days. Approximately 150 g of broilers feces were collected from each replicate on d 25, 26, 27 and 28. The fecal coccidia oocysts were counted d 25–28 after the feces collected every day were mixed thoroughly; then, 2 g feces from each sample was added to 58 mL saturated salt water, mixed thoroughly with a vortex shaker for 2 min, and sieved through a 40-mesh screen. The upper suspension was then added to a McMaster counting plate (Beijing Yikeran Biotechnology Co., Ltd., Beijing, China) and allowed to stand for 3 min. Oocysts were counted under a light microscope (Leica DM750), and the number of oocysts per gram of feces was calculated according to the following formula: oocyst content per gram of feces (opg) = number of oocysts in the counting chamber × 200 × dilution ratio. The opg per gram of feces was calculated after being log_10_-transformed and counted.

Cecal *C. perfringens* was counted as previously described by Wu et al. [11]. Approximately 0.5 g of each sample was collected in 10-mL sterile plastic tubes, diluted with PBS to an initial 10^−1^ dilution, and serially diluted to prepare 10^−1^ to 10^−5^ dilutions. Each diluted sample of 100 μL was plated on tryptose-sulfite-cycloserine agar (TSC, CM 138; Beijing Land Bridge Technology Co., Ltd., Beijing, China). The number of *C. perfringens* was determined after anaerobic incubation at 37 °C for 24 h. The *C. perfringens* was then log_10_-transformed and counted.

### Intestinal morphological analyses and observation

The middle ileum was circumcised to a thickness of 5 μm and stained with hematoxylin and eosin. The height of the villi and depth of the crypt of the chicken ileum were measured according to the method described by Frankel et al. [13]. Briefly, ten straight and structurally intact intestinal villi were selected from each intestinal section and measured. The mean value of each index was calculated to determine the ratio of villus height to crypt depth (V/C).

### Determination of serum immunoglobulin, C-reactive protein, myeloperoxidase, and ileal mucosa sIgA levels

The levels of natural serum IgA and IgM antibodies were determined using a chicken IgA enzyme-linked immunosorbent assay (ELISA) kit (SEKCN-0018, Beijing Solarbio Science & Technology Co., Ltd., Beijing, China) and chicken IgM ELISA kit (SEKCN-0128, Beijing Solarbio Science & Technology Co., Ltd.), respectively. C-reactive protein (CRP) (YJ036965, Shanghai Enzyme-linked Biotechnology Co., Ltd., Shanghai, China) and myeloperoxidase (MPO) kits (YJ966521, Shanghai Enzyme-linked Biotechnology Co., Ltd.) were used to determine the serum levels of CRP and MPO, respectively. The homogenized tissue was prepared by taking 0.1 g of ileal mucosa at a weight: saline volume ratio of 1:9, and the supernatant was retained for subsequent analysis. The sIgA and total protein contents in the ileal mucosa were determined using a chicken sIgA ELISA kit (YM-A3724, Shanghai Yuanmu Biotechnology Co., Ltd., Shanghai, China) and a total protein quantification kit (Nanjing Jiancheng Bioengineering Institute, Nanjing, China), respectively. Serum immunoglobulin content was measured in mg/mL, and mucosal sIgA content was measured in μg/mg protein. The above-mentioned indexes were measured in strict accordance with the manufacturer's instructions.

The antibody levels of *C. perfringens* in the serum were detected using an improved ELISA method. The methods used followed those described by Wu et al. [11] with slight alterations. *C. perfringens* was cultured to a concentration of 10^8^ CFU/mL and centrifuged at 4,000 r/min for 10 min; the thalli were collected and cleaned with 0.01 mol/L sterile PBS solution 3 times. At the third wash, the thalli were diluted with PBS and subjected to intermittent ultrasound over 10 times in an ice bath. When the bacterial solution was turbid and consistent, 9 mL PBS solution was added and incubated for more than 2 h. The supernatant was absorbed into another sterilized centrifuge tube. Protein concentration was measured using the total protein quantitative kit (Nanjing Jiancheng Bioengineering Institute, Nanjing, China) according to the manufacturer's instructions. Approximately 100 μL bacterial lysate of *C. perfringens* (40 μg/mL) was added to the enzyme-labeled plate and incubated overnight at 4 °C. The 96-well plates were coated with 20 μg/mL albumin from bovine serum (BSA) and incubated overnight at 4 °C. After washing 5 times with 200 μL of 1% BSA dissolved in PBS containing 0.05% Tween (PBST), the plates were incubated with the serum (1:50 dilution) at 37 °C for 1.5 h. Then, the plate was washed and 100 μL diluted horseradish peroxidase-conjugated goat anti-chicken IgG (1:10,000; A30-104P, Bethyl Laboratories Inc., Montgomery, TX, USA) was added and incubated at 37 °C for 30 min. After washing 5 times with PBST, 100 μL 0.05% tetramethylbenzidine was added to the plates and incubated at 37 °C in the dark for 30 min. The reaction was terminated with 2 mol/L sulfuric acid. The absorbance at 450 nm was measured using the SepctraMax i3x Multi Mode Detection Platform (Molecular Devices, LLC, Sunnyvale, CA, USA).

### Serum zonulin and endotoxin (ET) detection

The venous blood of chicken wings was collected using common collection vessels (5 mL), and the serum was collected after centrifugation at 3,500 r/min and 4 °C for 10 min. Zonulin (F8139-A, Shanghai Fanke Industrial Co., Ltd., Shanghai, China) and Endotoxin detection kits (ml0122369-J, Shanghai Enzyme-linked Biotechnology Co., Ltd., Shanghai, China) were then used according to the manufacturer’s instructions.

### Quantitative reverse transcription-PCR to measure mRNA expression in the ileum

The ileal tissues were collected, placed in RNase-free freezing tubes, and stored at −80 °C. Tissue samples (100 mg) were placed in a 2-mL centrifuge tube, 1 mL of Trizol (Invitrogen Life Technologies, Carlsbad, USA) was added, and total RNA was extracted according to the manufacturer’s instructions. The purity of the extracted RNA was checked using a nucleic acid spectrophotometer (AG 22331, Eppendorf, Hamburg, Germany), and samples with OD_260_/OD_280_ greater than 1.8 were used for subsequent processing. The samples were reverse transcribed using a cDNA kit (Takara Biotechnology Co., Ltd., Beijing, China). The Applied Biosystems 7500 Fast Real-Time PCR System and SYBR Premix Ex Taq™ kit (Takara Biotechnology Co., Ltd., Beijing, China) were used to performed quantitative reverse transcription-PCR. All procedures were performed according to the manufacturer’s instructions. Genes were quantified using β-actin as an internal reference, and the results were statistically analyzed according to the method described by Fu et al. [14]. The primer sequences for genes are shown in Table 2.

**Table 2:** Sequences of the oligonucleotide primers used for quantitative reverse transcription-PCR^a^

Gene	Primer sequences(5ʹ→ 3ʹ)	NCBI serial number
*β-actin*	F-CAACACAGTGCTGTCTGGTGGTAC	NM_205518.1
	R-CTCCTGCTTGCTGATCCACATCTG	
Occludin	F-ACGGCAGCACCTACCTCAA	NM_205128.1
	R-GGGCGAAGAAGCAGATGAG	
*MUC2*	F-TCCCCTGTTGAGGGAGAACTT	XM_040673077.1
	R-AGTGGTTGTACCTTCGGTGC	
*ZO-1*	F-CTTCAGGTGTTTCTCTTCCTCCTC	XM_040680632.1
	R-CTGTGGTTTCATGGCTGGATC	
*PEPT1*	F-TACGCATACTGTCACCATCA	NM_204365.2
	R-TCCTGAGAACGGACTGTAAT	
*SGLT1*	F-GATGTGCGGATACCTGAAGC	NM_001293240.1
	R-AGGGATGCCAACATGACTGA	
*GLUT2*	F-CCGCAGAAGGTGATAGAAGC	NM_205129.1
	R-ATTGTCCCTGGAGGTGTT	
*I-FABP*	F-GAAGCAATGGGCGTGAATGTGATG	NM_001007923.1
	R-TTCGATGTCGATGGTACGGAAGTTG	
*IL-1β*	F-ACTGGGCATCAAGGGCTA	XM_046931582.1
	R-GGTAGAAGATGAAGCGGGTC	
*IL-6*	F-CGCCCAGAAATCCCTCCTC	NM_204628.2
	R-AGGCACTGAAACTCCTGGTC	
*TLR2*	F-GATTGTGGACAACATCATTGACTC	NM_001161650.3
	R-AGAGCTGCTTTCAAGTTTTCCC	
*TGF-β*	F-TCATCACCAGGACAGCGTTA	NM_001031045.3
	R-TGTGATGGAGCCATTCATGT	

*F* Upstream primer, *R* Downstream primers, *ZO-1* Zonula occludens-1, *PEPT1* Peptide-transporter 1, *SGLT1* Sodium glucose cotransporter 1, *GLUT2* Glucose transporter 2, *I-FABP* ﻿Intestinal fatty acid binding protein, *IL-1β* Interleukin-1β, *IL-6* Interleukin-6, *TLR2* Toll like receptor 2, *TGF-β* Transforming growth factor-β

^a^The primers were synthesized by Shanghai Shenggong Biotechnology Co., Ltd.

### Ileal microbiological analysis

The contents of middle ileal samples collected on d 28 were used for 16S sequencing analysis, according to the method described by Zhang et al. [15]. First, fecal microbial DNA was extracted using QIAamp Fast DNA Stool Mini Kit (Qiagen Company, Dusseldorf, Germany), and then the purity of the DNA was tested using 1% agarose gel electrophoresis. All procedures were performed according to the manufacturer’s instructions. Bacterial DNA was amplified using the following V3-V4 primers: 338 F (5ʹ-ACTCCTACGGGAGGCAGCA-3ʹ) and 806R (5ʹ-GGACTACHVGGGTWTCTAAT-3ʹ). In-machine sequencing was performed using HiSeq2500 PE250, and the sequences were analyzed by Beijing Nuohe Zhiyuan Bio-Information Technology Co., Ltd (Beijing, China). The Qiime software (Qiime2-2019.7, Nature Biotechnology) was used to generate species abundance tables at different taxonomic levels. Analysis was then performed to identify biomarkers that were statistically different between groups and α, β diversity. Venn plots and principal co-ordinates analysis (PCoA) plots were plotted using the cloud platform (Beijing Nuohe Zhiyuan Bio-Information Technology Co., Ltd., Beijing, China), and analysis of similarities (ANOSIM) and PICRUSt analyses were performed.

### Statistical analysis

The data were analyzed using SPSS 26.0 (SPSS, Inc., Chicago, IL, USA). All groups were compared using one-way analysis of variance (ANOVA) and Duncan’s multiple comparisons. The linear and quadratic contrasts (PC, PTA1, PTA2, PTA3 and PTA4) were assayed using the contrast statement. The results are shown as the mean ± standard error of mean (SEM). The mortality was assayed using the Chi-squared test. A *P* < 0.05 was considered statistically significant and a 0.05 ≤ *P* ≤ 0.1 was classified as a tendency. Using Pearson’s correlation coefficient to evaluate the correlation analysis of the 28-day-old broiler growth performance, immunity, intestinal barrier, and other indicators of microbiota.

## Results

### Growth performance

The growth performance data for the broiler chickens from the control and treatment groups used in this study are shown in Table 3. The effect of tannic acid addition to the chicken diet showed a quadratic relationship with body weight and F/G. Additionally, it significantly increased the body weight at d 19 in the PTA1–PTA4 groups (*P* < 0.05) and markedly reduced the F/G during d 1–19 in the PTA2 and PTA3 groups (*P* < 0.05), whereas the PC group showed a significant decrease in growth performance at d 28 and a significant increase in the F/G during d 1–28 and d 1–35 (*P* < 0.05). On d 28, the PTA3 group showed a marked improvement in body weight and decrease in the F/G compared with PC group (*P* < 0.05). The F/G was significantly lower in the PTA2 and PTA3 groups during d 1–35 compared with PC group (*P* < 0.05). In addition, we found no significant differences (*P* > 0.05) in mortality between treatment groups.

**Table 3:** Effects of tannic acid on the growth performance of broilers with NE

	**NC**	**PC**	**PTA1**	**PTA2**	**PTA3**	**PTA4**	***P*-value**^**1**^	**Linear**^**2**^	**Quadratic**^**2**^
Bod Weight, g									
d 19	745.1 ± 6.0^c^	759.8 ± 7.5^c^	794.3 ± 6.4^ab^	801.2 ± 6.2^ab^	815.1 ± 11.3^a^	791.1 ± 5.6^b^	< 0.001	0.002	0.001
d 28	1,486.6 ± 12.4^a^	1,396.2 ± 8.8^c^	1,423.4 ± 7.9^bc^	1,430.7 ± 15.0^bc^	1,460.1 ± 19.5^ab^	1,414.8 ± 15.2^c^	0.001	0.105	0.025
d 35	2,011.7 ± 29.7	1,970.3 ± 12.0	2,010.5 ± 25.0	2,036.2 ± 24.2	2,050.3 ± 13.8	2,043.1 ± 16.0	0.111	0.004	0.145
F/G									
d 1–19	1.330 ± 0.013^a^	1.340 ± 0.016^a^	1.310 ± 0.008^ab^	1.270 ± 0.024^b^	1.280 ± 0.018^b^	1.300 ± 0.016^ab^	0.013	0.055	0.012
d 1–28	1.390 ± 0.004^c^	1.440 ± 0.008^a^	1.410 ± 0.007^bc^	1.420 ± 0.005^ab^	1.410 ± 0.012^bc^	1.430 ± 0.005^a^	< 0.001	0.612	0.009
d 1–35	1.530 ± 0.011^b^	1.570 ± 0.002^a^	1.540 ± 0.010^ab^	1.530 ± 0.010^b^	1.530 ± 0.010^b^	1.540 ± 0.004^ab^	0.022	0.017	0.007
mortality, %	1.90 ± 1.23	4.76 ± 1.90	1.90 ± 1.23	0.95 ± 0.95	2.86 ± 1.98	1.90 ± 1.23	0.768	-	-

*n* = 7. Data are the mean ± SEM. *NC* no tannic acid treatment or NE infection, *PC* NE infection + 0 mg/kg tannic acid, *PTA1* NE infection + 250 mg/kg tannic acid, *PTA2* NE infection + 500 mg/kg tannic acid, *PTA3* NE infection + 750 mg/kg tannic acid, *PTA4* NE infection + 1,000 mg/kg tannic acid

^1^Body weight and F/G P values obtained from ANOVA; the *P*-value of mortality was obtained by chi-square test; ^2^*P*-values obtained using contrast trend analysis. ^a^^–^^c^Different lower case letters in the same column indicate significant differences (*P* < 0.05)

### Intestinal morphology, lesion scores, and determination of cecal *C. perfringens* concentration

Lesion scores were performed on all duodenum, jejunum, and ileum after intestinal dissection. The results of the intestinal lesion scoring are shown in Table 4. We found that duodenum, jejunum, and ileum of chickens had different degrees of lesions. The lesions were mainly manifested in jejunum and ileum. While only a few chickens in the NC group showed slight intestinal congestion, all the chickens in the PC group showed gross macroscopic lesions. These included the thinning of the intestinal wall, flatulence, and localized bleeding. The addition of tannic acid improved the intestinal lesions, with a significant reduction in the ileal lesion score on d 28 in the PTA2 group (*P* < 0.05). In this study (Table 4), NE infection significantly increased the number of *C. perfringens* in the cecums of broilers (*P* < 0.05). According to the statistical results, the addition of tannic acid had no significant effect on the content of *C. perfringens* in cecum. However, from the numerical analysis, the addition of tannic acid has a certain inhibitory effect on *C. perfringens*. In particular, the number of *C. perfringens* in PTA3 group was about ten times lower than that in PC group.

**Table 4:** Intestinal lesion scores and cecum *C. perfringens* concentrations in broilers with NE on d 28

	**NC**	**PC**	**PTA1**	**PTA2**	**PTA3**	**PTA4**	***P*-value**^**1**^	**L**^**2**^	**Q**^**2**^
Intestinal lesion									
Duodenum	0.36 ± 0.18	0.50 ± 0.19	0.57 ± 0.17	0.36 ± 0.18	0.43 ± 0.13	0.10 ± 0.07	0.407	0.064	0.383
Jejunum	0.21 ± 0.15^b^	0.86 ± 0.09^a^	0.86 ± 0.09^a^	0.65 ± 0.09^a^	0.71 ± 0.10^a^	0.50 ± 0.15^ab^	0.003	0.018	0.693
Ileum	0.21 ± 0.15^bc^	0.57 ± 0.17^ab^	0.71 ± 0.15^a^	0.14 ± 0.07^c^	0.36 ± 0.09^abc^	0.29 ± 0.10^bc^	0.024	0.023	0.443
Cecum *C. perfringens*	2.75 ± 0.87^b^	6.00 ± 0.10^a^	5.44 ± 0.34^a^	5.37 ± 0.15^a^	5.33 ± 0.10^a^	5.17 ± 0.18^a^	0.001	0.007	0.262

*n* = 7. Data are the mean ± SEM. *NC* no tannic acid treatment or NE infection, *PC* NE infection + 0 mg/kg tannic acid, *PTA1* NE infection + 250 mg/kg tannic acid, *PTA2* NE infection + 500 mg/kg tannic acid, *PTA3* NE infection + 750 mg/kg tannic acid, *PTA4* NE infection + 1,000 mg/kg tannic acid, *L* Linear, *Q* Quadratic

^1^Overall *P*-values obtained from ANOVA; ^2^*P*-values obtained using contrast trend analysis. ^a^^–^^c^Different lower-case letters in the same column indicate significant differences (*P* < 0.05)

**Fig. 1:**
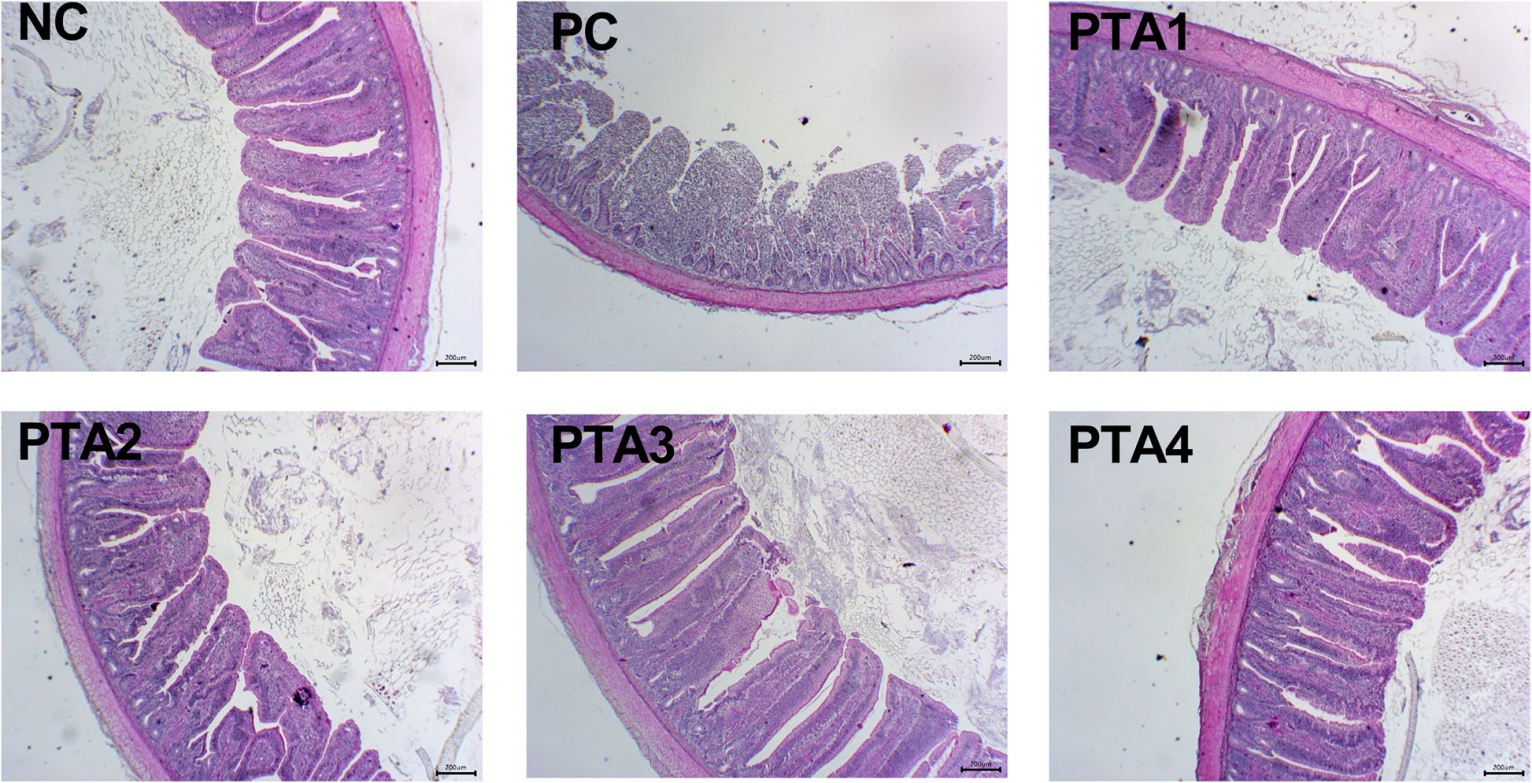
Representative photomicrographs of the cross sections of the middle ileum from chickens at d 28. The NC group shows normal physiological features of the intestinal villi, whereas the PC group shows severe pathological changes in the villi structure. The PTA1–PTA4 groups show mild pathological changes. NC: no tannic acid treatment or NE infection; PC: NE infection + 0 mg/kg tannic acid; PTA1: NE infection + 250 mg/kg tannic acid; PTA2: NE infection + 500 mg/kg tannic acid; PTA3: NE infection + 750 mg/kg tannic acid; PTA4: NE infection + 1,000 mg/kg tannic acid. The scale is 200 μm

Broilers in the NC group showed normal intestinal appearance, while those in the PC group showed obvious pathological changes in the villi, that were severely damaged, and the normal structure was destroyed. Intestinal lesions were alleviated by the addition of tannic acid to the feed (Fig. 1). The ileal villus height and V/C value significantly decreased in the PC group on d 28 (*P* < 0.05; Table 5). On d 28, compared with PC group, the ileal villus height was markedly increased in the PTA2–PTA4 groups (*P* < 0.05). In addition, compared with PC group, the V/C value significantly increased in the PTA1–PTA4 groups on d 28 (*P* < 0.05). Seven days after the infection, the ileal crypt depth was still markedly increased, and V/C decreased in the PC group (*P* < 0.05; Table 5). Compared with PC group, the ileal V/C values of the PTA1–PTA4 groups were significantly increased (*P* < 0.05) and the crypt depth of the PTA1–PTA4 groups were significantly reduced (*P* < 0.05), but neither NE nor tannic acid treatment had any effect on the ileal villus height on d 35.

**Table 5:** Effects of tannic acid on the morphology of the ileal midsection of broiler chickens with NE

	**NC**	**PC**	**PTA1**	**PTA2**	**PTA3**	**PTA4**	***P*-value**^**1**^	**L**^**2**^	**Q**^**2**^
d 28									
Vill height, μm	725.53 ± 21.88^ab^	627.54 ± 13.31^c^	661.86 ± 27.71^bc^	754.23 ± 28.68^a^	769.97 ± 17.58^a^	753.26 ± 23.13^a^	< 0.001	< 0.001	0.045
Crypt depth, μm	108.14 ± 3.55	123.09 ± 5.59	107.23 ± 4.13	113.97 ± 4.86	106.97 ± 2.87	109.87 ± 2.67	0.063	0.052	0.139
V/C	6.72 ± 0.17^ab^	5.15 ± 0.24^d^	6.18 ± 0.15^c^	6.64 ± 0.20^bc^	7.21 ± 0.14^a^	6.86 ± 0.14^ab^	< 0.001	< 0.001	< 0.001
d 35									
Vill height, μm	768.86 ± 44.27	738.36 ± 56.62	753.24 ± 31.83	739.62 ± 28.06	711.84 ± 15.71	734.47 ± 28.10	0.924	0.658	0.992
Crypt depth, μm	108.26 ± 4.29^b^	130.62 ± 7.04^a^	110.80 ± 2.86^b^	102.90 ± 5.17^b^	99.31 ± 2.95^b^	104.97 ± 2.92^b^	< 0.001	< 0.001	0.003
V/C	7.08 ± 0.22^a^	5.67 ± 0.35^b^	6.79 ± 0.17^a^	7.24 ± 0.23^a^	7.20 ± 0.24^a^	7.00 ± 0.20^a^	< 0.001	< 0.001	0.002

*n* = 7. Data are the mean ± SEM. *NC* no tannic acid treatment or NE infection, *PC* NE infection + 0 mg/kg tannic acid, *PTA1* NE infection + 250 mg/kg tannic acid, *PTA2* NE infection + 500 mg/kg tannic acid, *PTA3* NE infection + 750 mg/kg tannic acid, *PTA4* NE infection + 1,000 mg/kg tannic acid, *L* Linear, *Q* Quadratic

^1^Overall* P*-values obtained from ANOVA; ^2^*P-*values obtained using contrast trend analysis. ^a^^–^^c^Different lower-case letters in the same column indicate significant differences (*P* < 0.05)

### Fecal coccidia oocyst counts

**Fig. 2:**
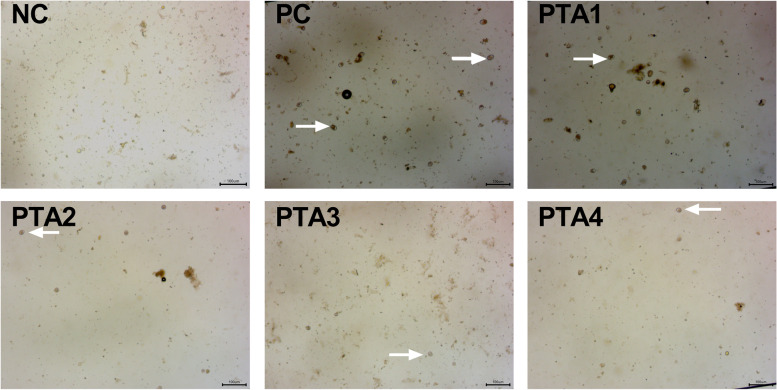
Representative picture of the coccidia oocysts in the feces at d 28. No coccidia oocysts were found in the feces of the NC group. The number of coccidia oocysts gradually decreased with an increase of tannic acid additive levels. NC: no tannic acid treatment or NE infection; PC: NE infection + 0 mg/kg tannic acid; PTA1: NE infection + 250 mg/kg tannic acid; PTA2: NE infection + 500 mg/kg tannic acid; PTA3: NE infection + 750 mg/kg tannic acid; PTA4: NE infection + 1,000 mg/kg tannic acid. The arrow indicates the coccidia oocysts

The coccidia oocyst counts are shown in Table 6. The coccidia oocysts were not observed in the NC group (Fig. 2). Addition of tannic acid to the diet linearly reduced the number of fecal coccidia oocysts (*P* < 0.05), and the numbers were lower in the PTA2 group at d 26 (*P* < 0.05), PTA3 and PTA4 groups at d 26–28 (*P* < 0.05), than those in the PC group.

**Table 6:** Effects of tannic acid on coccidia oocysts number in feces of broilers with NE

	**PC**	**PTA1**	**PTA2**	**PTA3**	**PTA4**	***P*-value**^**1**^	**Linear**^**2**^	**Quadratic**^**2**^
d 25	4.69 ± 0.03	4.60 ± 0.06	4.59 ± 0.03	4.59 ± 0.06	4.49 ± 0.04	0.064	0.008	0.954
d 26	4.78 ± 0.04^a^	4.75 ± 0.02^a^	4.66 ± 0.02^b^	4.61 ± 0.03^b^	4.61 ± 0.01^b^	< 0.001	< 0.001	0.370
d 27	4.63 ± 0.03^a^	4.54 ± 0.02^ab^	4.56 ± 0.03^ab^	4.49 ± 0.03^bc^	4.43 ± 0.04^c^	0.001	< 0.001	0.747
d 28	4.52 ± 0.04^a^	4.50 ± 0.04^ab^	4.43 ± 0.02^abc^	4.41 ± 0.03^b^	4.39 ± 0.03^c^	0.027	0.002	0.789

*n* = 7. Data are the mean ± SEM. *NC* no tannic acid treatment or NE infection, *PC* NE infection + 0 mg/kg tannic acid, *PTA1* NE infection + 250 mg/kg tannic acid, *PTA2* NE infection + 500 mg/kg tannic acid, *PTA3* NE infection + 750 mg/kg tannic acid, *PTA4* NE infection + 1,000 mg/kg tannic acid

^1^Overall *P*-values obtained from ANOVA; ^2^*P-*values obtained using contrast trend analysis. ^a^^–^^c^Different lower-case letters in the same column indicate significant differences (*P* < 0.05)

### Immune and intestinal barrier function

The changes in the levels of immune indicators in the serum and intestinal mucosa are shown in Table 7. The results showed that on d 28, the levels of IgM and *C. perfringens*-specific antibodies IgY, CRP, and MPO in the serum and sIgA in the ileal mucosa were significantly higher in the PC group than in the NC group (*P* < 0.05). Contrastingly, compared with that in the PC group, the addition of tannic acid to the feed reduced the sIgA content in the ileal mucosa on d 28 (*P* < 0.05). The levels of *C. perfringens*-specific antibodies IgY, MPO were significantly lower in the PTA3 group than in the PC group on d 28 (*P* < 0.05), and the levels of *C. perfringens*-specific antibodies IgY, CPR and MPO were significantly lower in the PTA4 group than in the PC group on d 28 (*P* < 0.05). On d 35, the levels of CRP and MPO in the serum of the PC group were significantlyly higher than those in the NC group (*P* < 0.05). The addition of tannic acid had no considerable effect on the levels of immunoglobulins, *C. perfringens*-specific antibodies IgY in the serum, and sIgA in the ileal mucosa on d 35; however, the serum levels of CRP and MPO were significantly reduced (*P* < 0.05).

**Table 7:** Effects of tannic acid on the serum immune indices of chickens with NE

	**NC**	**PC**	**PTA1**	**PTA2**	**PTA3**	**PTA4**	***P*-value**^**1**^	**L**^**2**^	**Q**^**2**^
d 28									
IgA, mg/mL	0.28 ± 0.04	0.34 ± 0.05	0.29 ± 0.03	0.30 ± 0.02	0.33 ± 0.03	0.28 ± 0.011	0.630	0.305	0.851
IgM, mg/mL	0.08 ± 0.01^c^	0.16 ± 0.02^ab^	0.15 ± 0.01^ab^	0.18 ± 0.03^ab^	0.21 ± 0.02^a^	0.15 ± 0.01^b^	0.001	0.807	0.163
Serum-specific IgY antibody, OD 450 nm	2.79 ± 0.05^b^	2.92 ± 0.03^a^	2.87 ± 0.02^ab^	2.85 ± 0.05^ab^	2.79 ± 0.04^b^	2.78 ± 0.03^b^	0.090	0.003	0.637
CRP, μg/mL	6.53 ± 0.76^c^	13.97 ± 1.28^a^	12.64 ± 1.20^ab^	12.78 ± 0.70^ab^	11.50 ± 0.63^ab^	10.77 ± 1.04^b^	< 0.001	0.024	0.952
MPO, pg/mL	697.06 ± 65.48^c^	1,291.64 ± 99.90^a^	1,305.64 ± 109.51^a^	1,132.20 ± 86.60^ab^	1,008.71 ± 103.05^b^	968.49 ± 76.27^b^	< 0.001	0.004	0.872
sIgA, μg/mg prot	30.42 ± 3.58^b^	52.52 ± 8.21^a^	34.88 ± 3.78^b^	35.13 ± 4.43^b^	28.96 ± 3.19^b^	28.07 ± 2.76^b^	0.008	0.001	0.149
d 35									
IgA, mg/mL	0.38 ± 0.04	0.37 ± 0.05	0.36 ± 0.08	0.36 ± 0.08	0.35 ± 0.04	0.31 ± 0.03	0.972	0.497	0.737
IgM, mg/mL	0.08 ± 0.01	0.08 ± 0.02	0.07 ± 0.02	0.08 ± 0.04	0.06 ± 0.003	0.05 ± 0.05	0.903	0.332	0.724
Serum-specific IgY antibody, OD 450 nm	2.87 ± 0.04	2.88 ± 0.03	2.92 ± 0.02	2.88 ± 0.03	2.88 ± 0.02	2.90 ± 0.03	0.903	0.964	0.993
CRP, μg/mL	9.02 ± 0.65^c^	14.00 ± 0.36^a^	11.27 ± 0.66^b^	10.16 ± 0.70^bc^	9.48 ± 0.87^bc^	8.59 ± 0.80^c^	< 0.001	< 0.001	0.128
MPO, pg/mL	765.28 ± 50.28^c^	1,570.16 ± 46.42^a^	1,009.84 ± 60.30^b^	973.55 ± 65.72^b^	871.87 ± 53.66^bc^	852.16 ± 57.78^bc^	< 0.001	< 0.001	< 0.001
sIgA, μg/mg prot	33.02 ± 4.45	38.44 ± 4.10	36.82 ± 2.67	35.72 ± 4.90	31.56 ± 3.13	29.74 ± 1.27	0.531	0.046	0.792

*n* = 7. Data are presented as mean ± SEM. *NC* No tannic acid treatment or NE infection, *PC* NE infection + 0 mg/kg tannic acid, *PTA1* NE infection + 250 mg/kg tannic acid, *PTA2* NE infection + 500 mg/kg tannic acid, *PTA3* NE infection + 750 mg/kg tannic acid, *PTA4* NE infection + 1,000 mg/kg tannic acid, *L* Linear, *Q* Quadratic

^1^Overall* P*-values obtained from ANOVA; ^2^*P*-values obtained using contrast trend analysis. ^a^^–^^c^Different lower-case letters in the same column indicate significant differences (*P* < 0.05)

Serum zonulin and ET levels showed consistent results (Table 8). Necrotic enteritis significantly increased serum zonulin and ET levels (*P* < 0.05), while the addition of tannic acid significantly reduced serum zonulin and ET levels (*P* < 0.05). A combined analysis of the zonulin and ET indices showed that the addition of tannic acid improved intestinal barrier function and reduced intestinal permeability.

**Table 8:** Effects of tannic acid on serum zonulin and ET in broiler chickens with NE

	**NC**	**PC**	**PTA1**	**PTA2**	**PTA3**	**PTA4**	***P*-value**^**1**^	**L**^**2**^	**Q**^**2**^
d 28									
Zonulin, ng/L	1,173.49 ± 26.79^d^	1,618.44 ± 23.52^a^	1,470.91 ± 15.53^b^	1,342.39 ± 23.42^c^	1,308.53 ± 27.41^c^	1,307.13 ± 23.76^c^	< 0.001	< 0.001	< 0.001
ET, EU/L	89.04 ± 3.15^c^	120.41 ± 2.86^a^	105.59 ± 5.22^b^	100.74 ± 3.54^b^	96.05 ± 3.19^bc^	94.51 ± 2.92^bc^	< 0.001	< 0.001	0.060
d 35									
Zonulin, ng/L	1,115.82 ± 17.94^e^	1,570.35 ± 25.15^a^	1,446.91 ± 21.36^b^	1,378.46 ± 15.37^c^	1,316.14 ± 27.27^ cd^	1,265.03 ± 27.26^d^	< 0.001	< 0.001	0.100
ET, EU/L	86.11 ± 3.14^ cd^	115.91 ± 3.89^a^	99.22 ± 3.85^b^	102.75 ± 2.93^b^	81.83 ± 2.20^d^	94.44 ± 4.19^bc^	< 0.001	< 0.001	0.014

*n* = 7. Data are presented as mean ± SEM. *NC* No tannic acid treatment or NE infection, *PC* NE infection + 0 mg/kg tannic acid, *PTA1* NE infection + 250 mg/kg tannic acid, *PTA2* NE infection + 500 mg/kg tannic acid, *PTA3* NE infection + 750 mg/kg tannic acid, *PTA4* NE infection + 1,000 mg/kg tannic cid, *L* Linear, *Q* Quadratic

^1^Overall *P*-values obtained from ANOVA; ^2^*P-*values obtained using contrast trend analysis. ^a^^–^^e^Different lower-case letters in the same column indicate significant differences (*P* < 0.05)

### mRNA expression of intestinal barrier gene, *MUC2,* and nutrient transport carriers

**Fig. 3:**
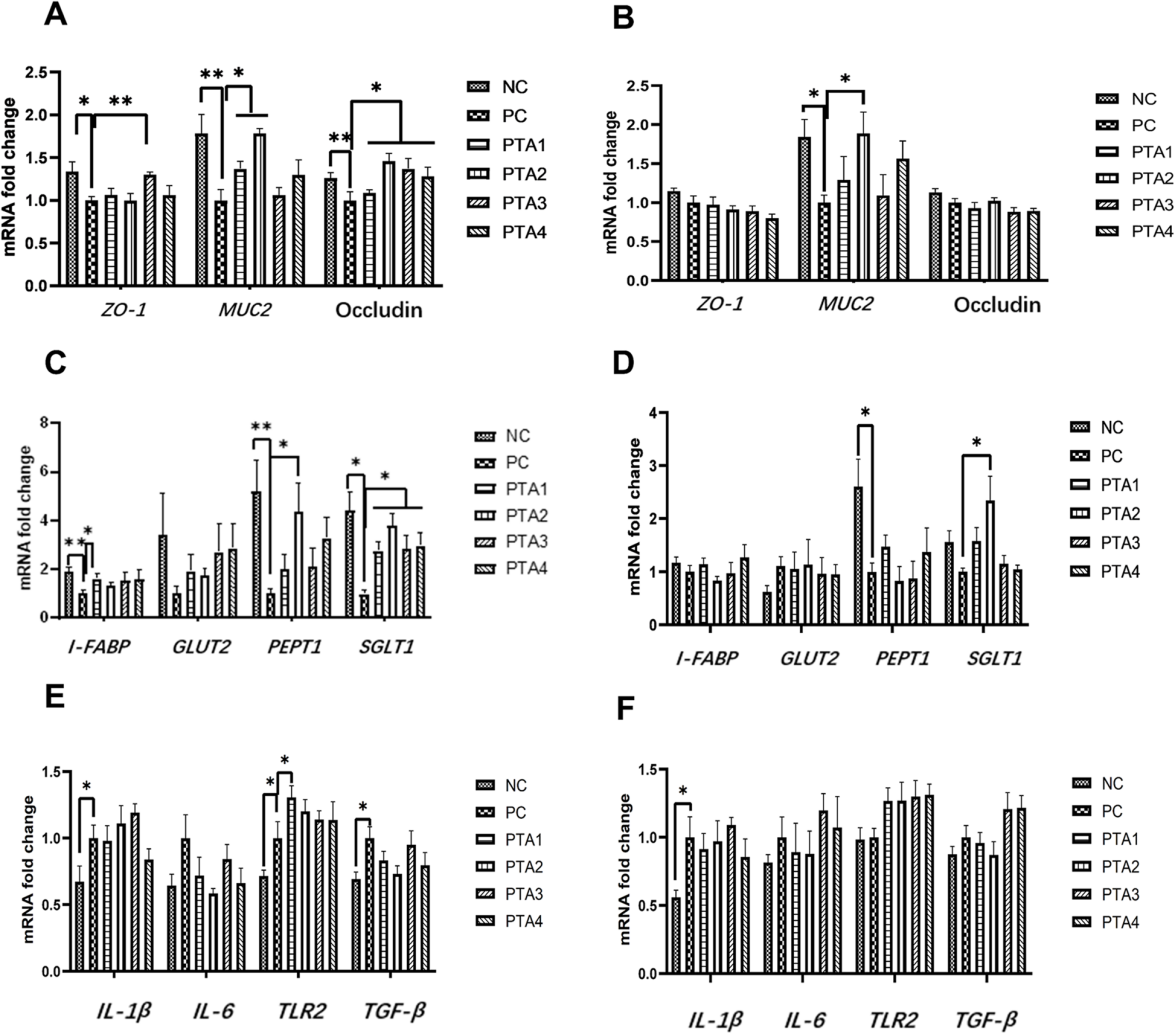
Effects of tannic acid on ileum gene transcription levels in broiler chickens with NE infection. **A** and **B** show the expression of genes related to the intestinal barrier and MUC2 in the ileum on d 28 and 35, respectively; **C** and **D** show the mRNA expression of genes related to nutrient transporter carriers in the ileum on d 28 and 35. **E** and **F** show the results of mRNA expression of genes related to immunity in the ileum on d 28 and 35. * and ** indicate 0.01 < *P* < 0.05 and *P* < 0.01, respectively. NC: no tannic acid treatment or NE infection; PC: NE infection + 0 mg/kg tannic acid; PTA1: NE infection + 250 mg/kg tannic acid; PTA2: NE infection + 500 mg/kg tannic acid; PTA3: NE infection + 750 mg/kg tannic acid; PTA4: NE infection + 1,000 mg/kg tannic acid. The error bars represent SEM. *n* = 7

The results of intestinal barrier gene and *MUC2* expression on d 28 (Fig. 3A) showed that the expression of *ZO-1* in the PC group was reduced (*P* < 0.05) and the mRNA expression of *MUC2* and Occludin was reduced (*P* < 0.01) compared to that in the NC group. The addition of tannic acid to the feed significantly increased the expression of Occludin compared with that in the PC group (*P* < 0.05); *MUC2* expression was increased in the PTA1 and PTA2 groups (*P* < 0.05), and *ZO-1* expression was significantly increased in the PTA3 group (*P* < 0.01). The results for intestinal barrier genes and *MUC2* mRNA expression on d 35 (Fig. 3B) showed that *MUC2* expression in the PC group was still lower than that in the NC group (*P* < 0.05). The expression of *MUC2* was higher in the PTA2 group than in the PC group (*P* < 0.05).

Regarding the ileal nutrient transport carriers on d 28 (Fig. 3C), the mRNA expression of intestinal *I-FABP* and *PEPT1* was significantly lower in the PC group (*P* < 0.01) and that of *SGLT1* was lower in the PC group (*P* < 0.05) than in the NC group. Compared with that in the PC group, the addition of tannic acid to the feed increased the expression of *SGLT1* in the PTA1–PTA4 groups (*P* < 0.05), *I-FABP* in the PTA1 group (*P* < 0.05) and *PEPT1* in the PTA2 group (*P* < 0.05). The ileal nutrient transport carrier results on d 35 showed that the expression of *PEPT1* in the PC group was lower than that in the NC group (*P* < 0.05; Fig. 3D). The relative mRNA expression of *SGLT1* was higher in the PTA2 group than in the PC group on d 35 (*P* < 0.05). Neither NE infection nor tannic acid addition affected the expression of *GLUT2*.

We found that the expression levels of *IL-1β*, *TLR2*, and *TGF-β* under NE conditions were increased on d 28 (*P* < 0.05; Fig. 3E). After tannic acid supplementation, *TLR2* mRNA expression levels in the PTA1 group were increased compared with those in the PC group (*P* < 0.05). Tannic acid supplementation had no significant effect on *IL-1β*, *IL-6*, *TGF-β* mRNA expression levels. On d 35, compared with those in the NC group, *IL-1β* mRNA expression levels were increased in the PC group (*P* < 0.05; Fig. 3F), but tannic acid supplementation had no significant effect on *IL-1β*, *IL-6*, *TGF-β* mRNA expression levels.

### Ileal microbiological analysis

**Fig. 4:**
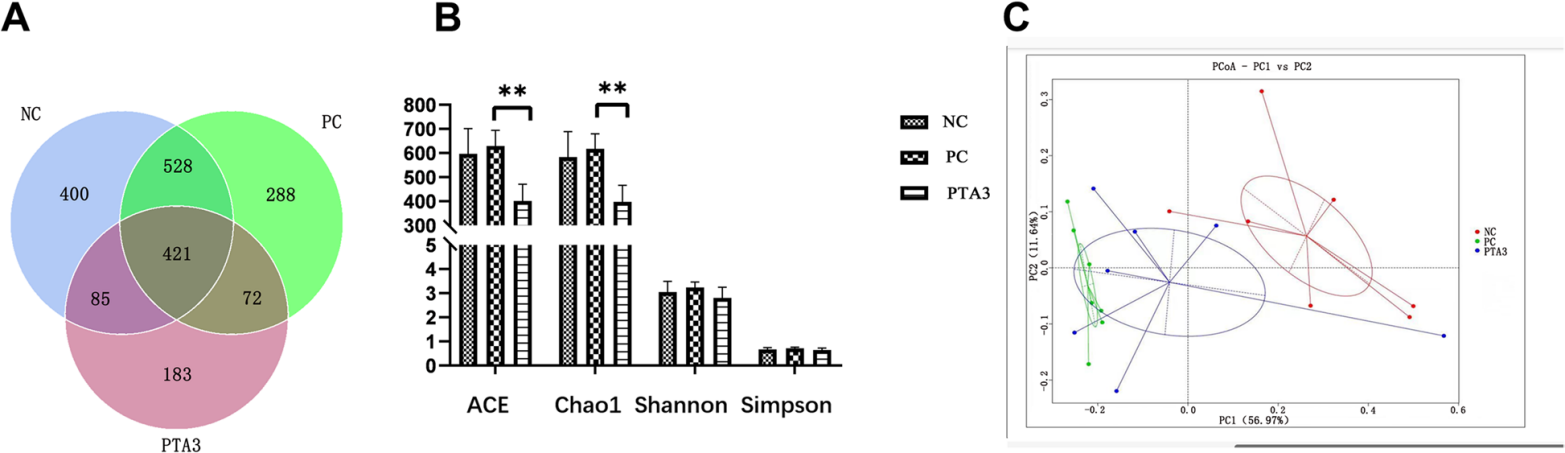
Effects of tannic acid on ileal microbial diversity in broiler chickens with NE. **A** Venn diagram showing the OTUs of the ileal microorganisms. **B** α-diversity diagram showing ileal microorganisms. **C** PCOA diagram. * and ** indicate 0.01 < *P* < 0.05 and *P* < 0.01, respectively. NC: no tannic acid treatment or NE infection; PC: NE infection + 0 mg/kg tannic acid; PTA3: NE infection + 750 mg/kg tannic acid. The error bars represent SEM. *n* = 7

To study the effect of tannic acid on the microbiota of the ileal midsection of broilers infected with NE, the changes in the ileal microorganisms were compared among the NC, PC, and PTA3 groups. There were 400 unique OTUs in the NC group, 288 in the NE group, and 183 in the PTA3 group (Fig. 4A). Alpha diversity was measured using the ACE, Chao1, Simpson, and Shannon indices. Figure 4B shows that NE did not significantly affect the microbial alpha diversity in mid-ileum microorganisms. The ACE and Chao1 indices were significantly reduced after the addition of tannic acid (*P* < 0.01), but there were no significant differences in the Shannon or Simpson indices. The principal component analysis (PCoA) of ileal microorganisms is shown in Fig. 4C. The principal component analysis showed there was a certain degree of dispersion between the three groups. The figure of PCoA combined with Table 9 showed that there were remarkable differences in the composition and structure of the ileal microbiota between these groups (*P* < 0.01).

**Table 9:** ANOSIM for flora composition among the three treatments

Treatment	*R* value	*P*-value
NC-PC	0.799	0.003
PC-PTA3	0.238	0.008

*R* values range from −1 to 1. Differences between groups were significant for *R* values > 0, and differences within groups were greater than those between groups for *R* values < 0. *P* < 0.05 indicates significant differences

**Fig. 5:**
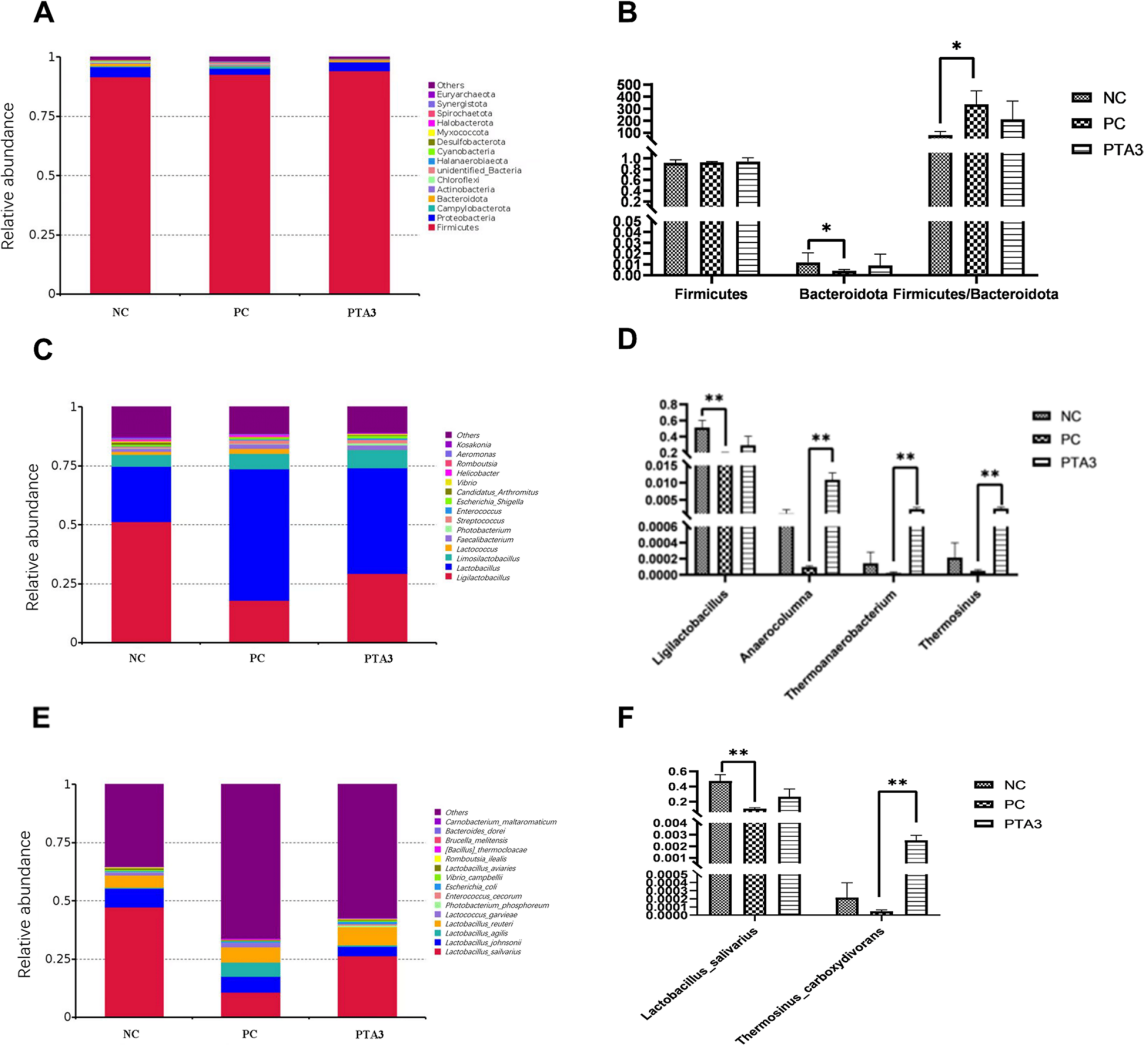
Effect of tannic acid on the relative abundance of microorganisms in the ileum of NE. **A**, **C**, and **E** The abundance of the top fifteen species at the phylum, genus, and species levels; **B**, **D**, and **F** The differential species at the phylum, genus, and species levels. * and ** indicate 0.01 < *P* < 0.05 and *P* < 0.01, respectively. NC: no tannic acid treatment or NE infection; PC: NE infection + 0 mg/kg tannic acid; PTA3: NE infection + 750 mg/kg tannic acid. The error bars represent SEM. *n* = 7

Figure 5A showed the top 15 species of phyla horizontal abundance. Firmicutes, Proteobacteria, Campylobacterota, and Bacteroidota occupied more than 95%. At the phylum level (Fig. 5B), NE infection reduced the relative abundance of Bacteroidota and increased the ratio of Firmicutes/Bacteroidota (*P* < 0.05), and no significant difference in Firmicutes was observed between the three groups. Figure 5C showed the top 15 species with the genus horizontal abundance, of which *Ligilactobacillus* and *Lactobacillus* occupied about 75%. At the genus level (Fig. 5D), *Ligilactobacillus* abundance was significantly reduced in the PC group (*P* < 0.01), and the addition of tannic acid significantly increased the relative abundance of *Anaerocolumna*, *Thermoanaerobacterium*, and *Thermosinus* (*P* < 0.01). Figure 5E showed the top 15 species of species horizontal abundance. The effects of challenge on the intestinal *Lactobacillus*-associated flora were more pronounced at the species level (Fig. 5F), with the abundance of *Lactobacillus salivarius* flora in the PC group significantly reduced (*P* < 0.01); addition of tannic acid significantly increased the relative abundance of *Thermosinus carboxydivorans* (*P* < 0.01).

### Prediction of bacterial flora function

**Fig. 6:**
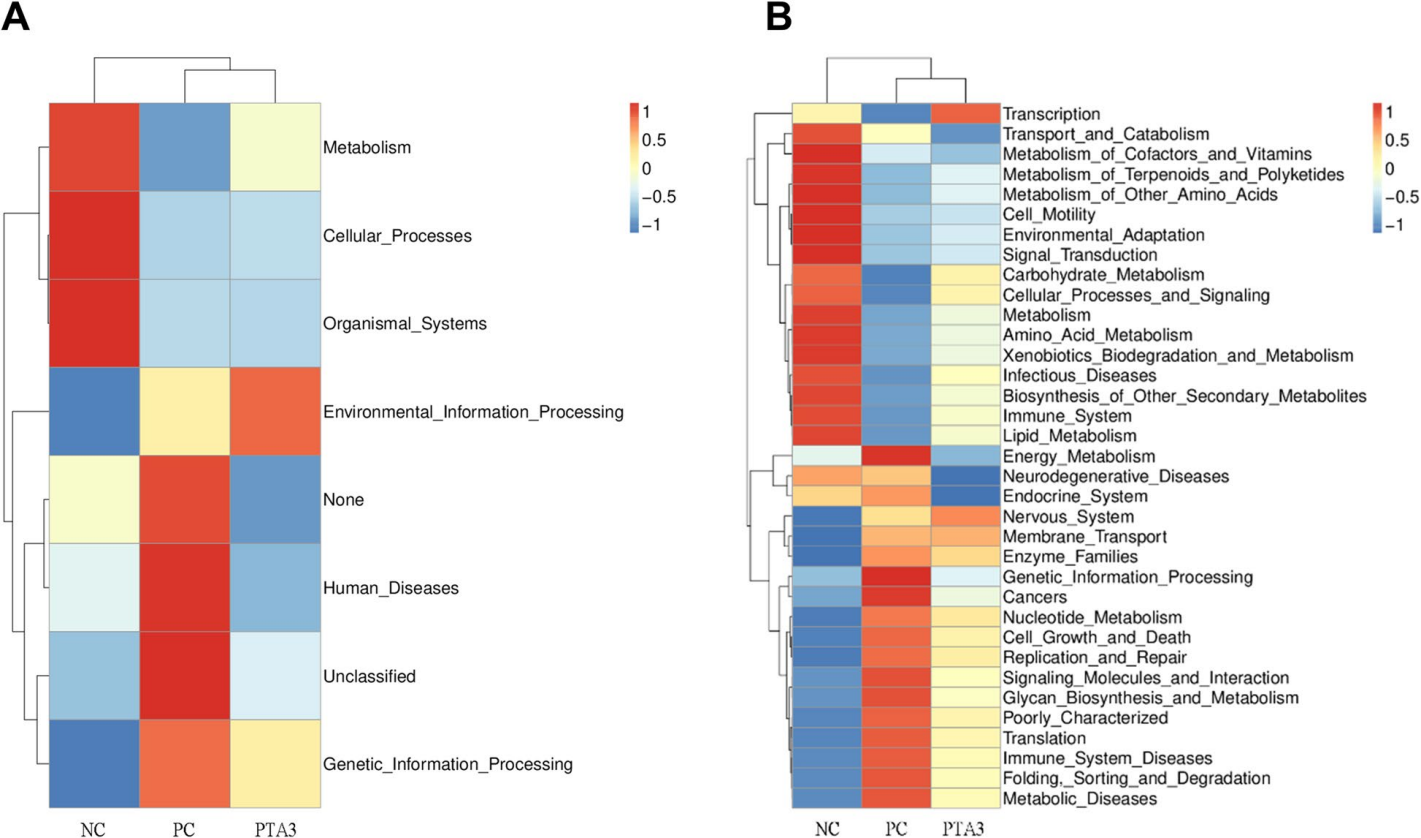
Effects of tannic acid on the microflora in the mid-ileum of broiler chickens with NE. Results of the functional clustering at (**A**) level 1 and (**B**) level 2. NC: no tannic acid treatment or NE infection; PC: NE infection + 0 mg/kg tannic acid; PTA3: NE infection + 750 mg/kg tannic acid. *n* = 7

PICRUSt analysis was performed online using the microbial genome information from the Kyoto Encyclopedia of Genes and Genomes (KEGG) (Fig. 6A and B). This analysis allowed for a comparison of the differences in functional profiles among the groups and revealed considerably different functional clustering of the gene pathways among the NC, PC, and PTA3 groups. The results showed that eight gene pathways differed among the three groups at level 1, mainly involving metabolism, cellular processes, environmental and genetic information processing and disease-related gene pathways. The gut microbes of the NC group were mainly enriched in biological systems, cellular processes, and metabolic pathways; those of the PC group were mainly enriched in disease and genetic information processing pathways, while those of the PTA3 group were mainly enriched in environmental information processing signaling pathways. The results showed 35 different gene pathways at level 2. Among them, NE infection considerably downregulated the metabolic pathways of carbohydrates, lipids, and amino acid. The addition of tannic acid upregulated the pathways related to the metabolism of these nutrients to some extent. Furthermore, we found that the metabolic pathways related to energy, nucleotides, cell growth and death, replication and repair, cancer, disease, and disease immunity were considerably elevated in the intestinal flora after challenge and that the addition of tannic acid downregulated these metabolic pathways to a certain extent.

### Indices correlation analysis

**Fig. 7:**
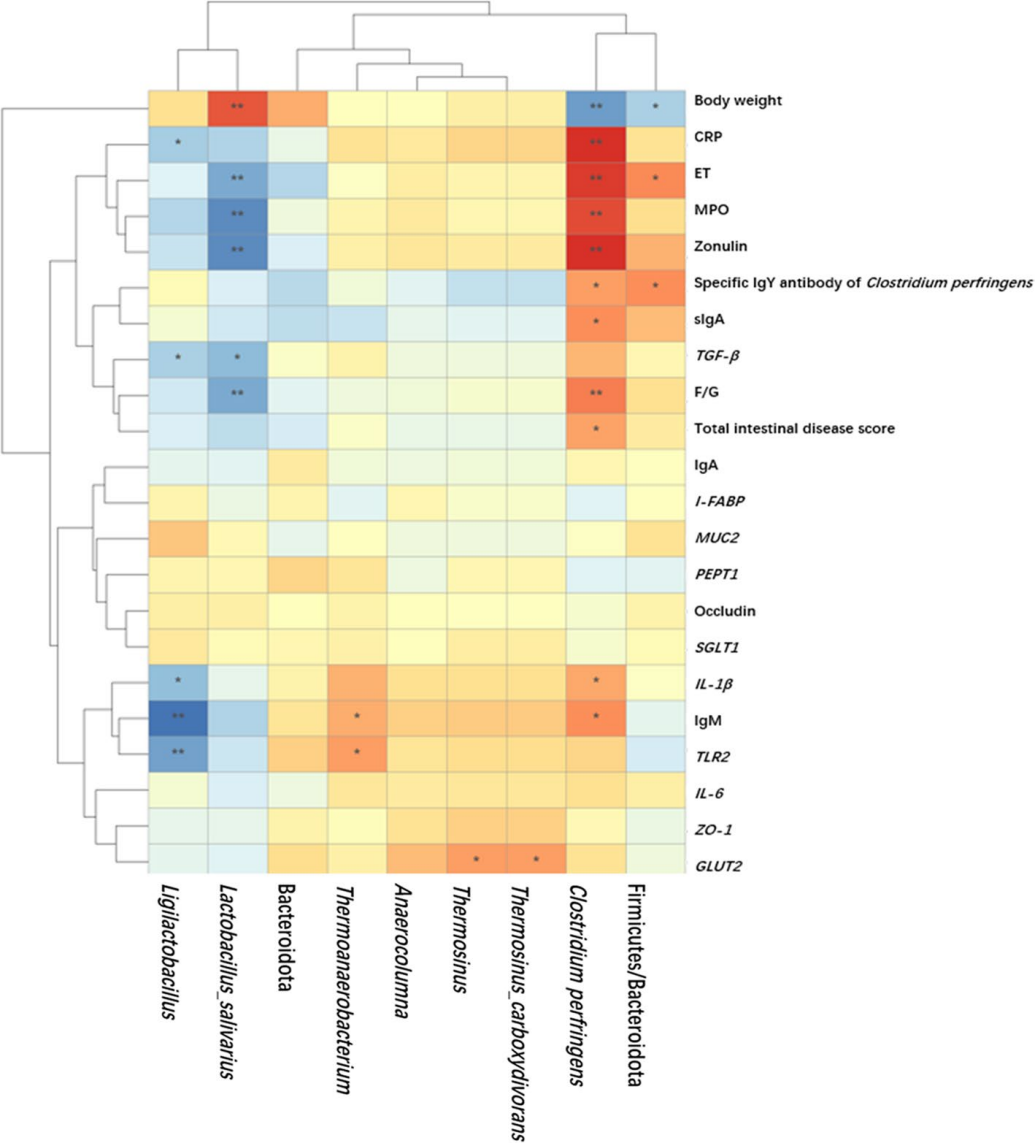
Correlation heat map for effect of tannic acid on the indices of broilers with NE. The graph demonstrates the Pearson correlation between the indicators. Squares are colored Pearson correlation coefficient *R* values. * and ** indicate 0.01 < *P* < 0.05 and *P* < 0.01, respectively

To explore the relationship between the effect of tannic acid on the gut microorganisms of broiler chickens with NE and the indices for growth performance and immunity, Pearson correlation analysis was conducted based on the above-shown microbial data. *Ligilactobacillus* showed a highly significant negative correlation with IgM levels in the serum and *TLR2* mRNA expression level (*P* < 0.01*,* Fig. 7); *Ligilactobacillus* showed a negative correlation with CRP, *TGF-β*, and *IL-β* levels (*P* < 0.05); *Lactobacillus salivarius* showed a highly significant positive correlation with body weight (*P* < 0.01) and a highly significant negative correlation with the F/G, ET, MPO, and zonulin levels (*P* < 0.01). In addition, *Lactobacillus salivarius* was negatively correlated with the mRNA expression level of *TGF-β* (*P* < 0.05). The Firmicutes/Bacteroidota ratio exhibited a negative correlation with body weight (*P* < 0.05) and a positive correlation with serum ET levels and *C. perfringens*-specific antibodies (*P* < 0.05); *Thermoanaerobacterium* showed a significant positive correlation with serum IgM and *TLR2* mRNA expression levels (*P* < 0.05); *Thermosinus carboxydivorans* and *Thermosinus* were significant positive correlation with *GLUT2* mRNA expression levels (*P* < 0.05). The number of *C. perfringens* in the cecum was significantly positively correlated with CRP, ET, MPO, and zonulin levels and the F/G ratio (*P* < 0.01) and positively correlated with IgY, sIgA, IgM, *IL-1β* and intestinal lesion score (*P* < 0.05). In addition, the number of *C. perfringens* was negatively correlated with body weight (*P* < 0.01).

## Discussion

In this study, we investigated the modulating effects of different tannic acid additive levels on the intestinal health of broiler chickens co-infected with coccidia and *C. perfringens*. Although NE did not cause a significant increase in mortality, NE infection was found to considerably reduce the body weight and increase the F/G of broilers, and their small intestines showed remarkable pathological changes, such as intestinal congestion, red bruises, thinning of the intestinal wall, and intestinal distension. The intestinal section analysis further showed damage to the villi structure in the PC group, which indicated the successful establishment of the necrotic enteritis (NE) model and was consistent with the results of previous studies [15, 16]. Regarding the intestinal barrier genes as well as nutrient transporters, the mRNA expression levels in the PC group was negatively affected. Earlier studies have reported that NE reduces growth performance and elevated serum ET [15], which is consistent with our findings. In addition, the serum levels of zonulin were elevated, further indicating the successful establishment of NE model. Tannic acid addition to the feed had beneficial effects on the broilers with NE and resulted in reduced intestinal lesion scores, increased villus height, and improved intestinal morphology.

### Effects of tannic acid addition to the diet on the growth performance of broiler chickens with NE

Results showed that tannic acid in the feed could ameliorate the growth performance degradation caused by NE, which is consistent with the results of previous studies. Yang et al. [17] observed better F/G ratios when broilers were fed 7.5 to 15 mg/kg proanthocyanidins. Wang et al. [18] investigated the effects of addition of 5–80 mg/kg grape seed extracts to the coccidia-challenged diet and found that 10–20 mg/kg markedly improved the growth performance; these results were consistent with those of this study. However, some studies have shown that adding 20 g/kg tannic acid considerably reduced body weight gain and the feed conversion efficiency in broilers [10]. Jamroz et al. [19] added 0.025%–0.1% sweet chestnut tannin to the diet and found that < 0.05% had no effect on growth performance, whereas 0.1% notably reduced growth performance. These findings suggest that the biological effects of tannins are highly dose-dependent, and the inconsistent effects of tannins in the literature may be due to differences in their source, dose, extraction process, encapsulation, and basal diets. In the present study, the growth performance showed a quadratic curve that increased with the addition of tannic acid; 500–750 mg/kg tannic acid was found to be the optimal concentration for improving the growth performance. The reason dietary tannic acid improves the growth performance of NE may be that it enhances the intestinal barrier, improves intestinal morphology, balances microbiota, and improves nutrient absorption capacity.

### Effects of tannic acid on the intestinal morphological structures of broiler chickens with NE infection

A normal villus morphological structure is a prerequisite for the intestine to perform its absorption function. An increase in villus height may lead to enhanced nutrient absorption, and a lower crypt depth indicates a decrease in the metabolic cost of intestinal epithelial renewal. Our findings suggested that NE considerably increased crypt depth, indicating that there was faster tissue renewal for the villi, as needed in response to the inflammation caused by pathogens or their toxins, which is consistent with previous findings [11, 20]. The addition of tannic acid notably reduced the increase in the depth of intestinal crypts caused by NE and improved the V/C ratio, demonstrating the beneficial effects of tannic acid on the intestinal morphology. The V/C showed a quadratic curve change with the addition of tannic acid, which is consistent with our results regarding the growth performance, as 500–1000 mg/kg tannic acid was highly effective at ameliorating the intestinal morphology.

### Effects of tannic acid on the fecal coccidia oocysts excretion and cecum *C. perfringens* in broiler chickens with NE infection

Examining coccidia oocyst shedding is an effective method to determine the level of coccidia infection [21]. The addition of tannic acid reduced the number of coccidia oocysts shed compared to that in the PC group, which is consistent with the results of previous reports [18, 22, 23]. The reduction in the number of oocysts in the feces indicated an increase in coccidia resistance in broilers. In the present study, the amount of excreted coccidia oocysts decreased linearly with an increase in tannic acid concentration. The decrease in the number of coccidia oocysts after tannic acid addition could be attributed to the modulation of intestinal microorganisms by tannic acid and its own antiparasitic biological properties [24, 25]. Taken together, tannic acid supplementation reduced coccidia proliferation, markedly reduced the number of coccidia oocysts excreted in feces, accelerated coccidia clearance, and improved intestinal health.

It was also determined that tannic acid linearly reduced the number of *C. perfringens* in cecum, especially PTA4 group as it had the most obvious inhibition effect on *C. perfringens*, which was about ten times less than PC group. The reasons for the reduction of *C. perfringens* content in the cecum may be due to tannic acid forming a thin layer of insoluble and denatured protein in the digestive tract, that covers the mucosal surface of the intestinal wall and reduces the colonization of *C. perfringens* on the intestinal mucosa. The complex reaction of tannic acid with biomacromolecules and the chelation of metal ions are the basis of its bacteriostatic effect. Tannic acid may alter the permeability and physiological function of the cell membrane by interacting with the membrane and extracellular proteins (e.g., metal ions, enzymes) of *C. perfringens*.

### Effects of tannic acid on the immune indices in broiler chickens with NE

The addition of tannic acid also aided in the alleviation of impaired intestinal morphology and elevated lesion scores caused by NE infections. This process was accompanied by changes in the serum levels of IgM, MPO, CRP and *C. perfringens*-specific antibodies in broilers. IgM is a circulating antibody secreted by B cells that first responds to initial encounters with foreign antigens; however, the IgM concentrations in the blood rapidly declined due to clearance, which is consistent with our study findings, as IgM levels were found to be elevated during the first days of infection with NE and did not change considerably 7 d after the infection. C-reactive protein is an acute temporal protein that activates complement and enhances phagocytosis, and MPO is mainly found in myeloid cells; both are markers of the inflammatory response. In this study, the increased MPO and CRP activities in the serum of the PC group indicated that NE infection could activate immune cells in the blood and promote inflammation. The addition of tannic acid to the feed reduced the inflammatory response caused by NE and the concentration of *C. perfringens*-specific antibodies in the serum, which may be related to the anti-inflammatory and *C. perfringens* proliferation inhibitory properties of tannic acid [4, 7]. The results of this experimental study showed that NE infection exerted long-term effects on broiler chickens, including reduced intestinal barrier function 7 d after infection, continued high levels of MPO and CRP in the serum, and inferior growth performance compared to those in the NC group.

In innate immunity, the intestinal mucosa is considered the first line of defense against pathogen infection, and mucosal immunity plays an important role in NE. The results of the present study showed that NE infection considerably damaged the ileum and stimulated the expression of sIgA in the intestinal mucosa, which is consistent with the results of an earlier study [11]. We found that tannic acid reduced the sIgA secretion in the ileal mucosa induced by NE, which might be because tannic acid reduced the number of coccidian merozoites and the colonization of *C. perfringens*. In addition, we observed that *TLR2* was activated, and inflammatory factor expression was increased in NE, consistent with previous studies [26, 27]. The activation of *TLR2* subsequently activates the downstream signaling pathways, on the one hand, and by inhibiting the number of *C. perfringens* and coccidia, on the other hand, thus reducing the inflammatory response.

### Effects of tannic acid addition to the feed on mRNA expression of the gut barrier genes and nutrient transport carriers in chickens with NE

The intestine plays a key role in the defense against pathogens, host nutrient digestion, and absorption [28]. Numerous studies have shown that the mRNA expression of tight junctions and nutrient transport carriers decreased and nutrient digestion and absorption are diminished during intestinal inflammation [29–32]. Intestinal inflammation results in a reduction in mucin synthesis and the number of cupped cells, therefore increasing the chances of further intestinal infection and bacterial translocation. In this study, NE led to a reduction in the mRNA expression of Occludin, *ZO-1*, and *MUC2*, causing an increase in intestinal permeability. However, the mRNA expression of intestinal tight junctions and *MUC2* was increased with the addition of tannic acid compared to that in the PC group, indicating the positive effects of tannic acid in protection of chickens from NE.

Zonulin is the only known regulator of tight intercellular junctions and is involved in the regulation of intestinal barrier function. Studies have also shown that zonulin levels in the serum are remarkably elevated in patients with irritable bowel syndrome or Crohn’s disease [33]. However, the changes in the sensitivity to zonulin in poultry intestinal diseases require further investigation. In the present study, NE led to a notable increase in zonulin levels in the serum, indicating that the intestinal barrier permeability was increased. Endotoxin is a Gram-negative bacterial cell wall component, and serum ET levels are elevated in broiler chickens suffering from NE [34, 35], which was also confirmed in our study. The addition of tannic acid to the feed increased the mRNA expression of Occludin, *ZO-1*, and *MUC2* and improved the integrity of the intestinal barrier, thereby reducing zonulin and ET levels. Nevertheless, the upregulation of intestinal tight junction-related pathways by tannic acid requires further investigation.

I-FABP is a cytoplasmic protein located in mature cells at the top of intestinal villi, and it plays an important role in fatty acid uptake and metabolism. We observed that the mRNA expression of *I-FABP* was reduced in broiler chickens infected with NE, which was consistent with the results of an earlier study [36]. However, it has also been shown that the mRNA expression of *I-FABP* does not change considerably in the dexamethasone-induced intestinal disorder model or infection with *Campylobacter jejuni* [37, 38], which might be due to the different disease models and severity. In addition, we observed a decrease in the mRNA expression of *PEPT1* and *SGLT1* in broiler chickens infected with NE, which was consistent with the results of an earlier study [39]. The addition of tannic acid improved the mRNA expression of nutrient transporter carriers and reduced the negative effects of NE on nutrient absorption in chickens. A comprehensive analysis revealed that growth performance, intestinal villus height, crypt depth, V/C, and nutrient transporter expression showed relatively consistent quadratic curve changes after tannic acid was added to the diet. The reason for this is that lower doses of tannic acid can reduce gastrointestinal peristalsis, thereby increasing the residence time of chyme in the gastrointestinal tract and improving nutrient digestibility and intestinal health. However, high doses of tannic acid exert a negative impact on growth performance by binding starch and protein in the feed, reducing digestive enzyme activity, and binding to intestinal wall proteins [40, 41].

### Effects of tannic acid on the ileal microflora of broilers with NE

The intestinal microbiota forms a highly complex microecosystem. To further investigate the mechanisms by which tannic acid alleviates the intestinal damage caused by NE, we analyzed the microflora structure of the ileal microorganisms using 16S rRNA sequencing. The results of this study showed that the challenge did not have a significant effect on the microbial α-diversity, which is consistent with the results of previous studies [42, 43]. We speculated that the possible reason for this result was that NE infection inhibited the proliferation of minor microorganisms in the broiler intestinal flora, which resulted in a convergence with the α-diversity of the NC group microbiota. However, NE has also been shown to result in either lower or higher gut microbial α-diversity [15, 44], which may be related to the collection of different parts of the chyme. In the present study, addition of tannic acid notably reduced the α-diversity but did not affect the balance abundance of the flora, which was consistent with a previous study [45]. It has been shown that low-abundance microbes lead to the formation of simpler metabolic networks, resulting in increased concentrations of specific components used to support host energy requirements [45]. Shabat et al. [46] reported that a reduced microbiome abundance may be closely associated with an increased feed conversion efficiency, with specific enrichment and metabolic pathways of microbes leading to better energy and carbon delivery to the animal body, which could be one of the reasons tannic acid improves growth performance. The significant difference in β-diversity among the three groups indicated that the addition of tannic acid or challenges significantly altered the microbial community structures. Bacteroidota are an important contributor to intestinal health, and this phylum plays an important role in breaking down complex molecules into simpler compounds and producing short-chain fatty acids that are beneficial for increased growth performance [47, 48]. We found that NE led to a decrease in the abundance of Bacteroidota, which is consistent with the results of a previous study [43]. The fermentation products of Bacteroidota, such as *Bacteroides fragilis*, inhibit *C. perfringens* spore formation, and the decrease in Bacteroidota may predispose the animal intestine to *C. perfringens* infection and gastroenteritis [49], which may be one of the reasons for the decrease in Bacteroidota abundance in NE. Lately, several studies have suggested that stressed birds present a notable increasing trend for the Firmicutes/Bacteroidota ratio [50, 51]. In this study, we found that the high Firmicutes/Bacteroidota ratio caused by NE may be one of the reasons for dysbiosis of the gut microbial community.

The results also showed that at the genus level, NE considerably reduced the abundance of the beneficial bacterium *Ligilactobacillus*, suggesting that NE infection inhibited the growth of beneficial intestinal bacteria. At the species level, NE notably reduced the relative abundance of *Lactobacillus salivarius*, which is a probiotic in poultry that dates back more than 15 years. Furthermore, this bacterium can re-establish the proper microbial balance of bacteria by forming lactic and propionic acid, stimulate butyric acid-producing butyric acid production, and inhibit the production of pro-inflammatory cytokines [52]. In broilers, *Lactobacillus salivarius* was found to improve growth performance, reduce the expression of inflammatory factors, and improve intestinal health [9, 53, 54]. In conclusion, we found that the intestinal flora of broiler chickens infected with NE was disturbed and the abundance of beneficial bacteria was reduced, leaving the intestine in an unhealthy state. The addition of tannic acid after the challenge considerably increased the relative abundance of the genus *Anaerocolumna*, which belongs to the *Lachnospiraceae* and can be metabolized by the fermentation of carbohydrates into acetate [55]. In addition, the relative abundance of *Thermoanaerobacterium*, which can secrete xylanase, degrade hemicellulose and even cellulose, and produce acetate and butyric acid via fermentation using xylan and others, was increased [56]. The genus *Thermosinus* includes Gram-negative bacteria that can convert CO to carbon dioxide through a series of chemical reactions and can decompose organic substrates (glucose, sucrose, or lactose) to produce acetic acid as well as acetate, H_2_, and CO_2_ during glucose fermentation [57]. Thus, it regulates the intestinal pH, nourishes butyric acid-producing bacteria, protects against pathogens, and contributes to intestinal health. In conclusion, the addition of tannic acid increases the abundance of short-chain fatty acid-producing-related flora, which may be one of the reasons for their beneficial impacts on intestinal health.

In the PICRUSt analysis, we found that differences in the metabolic pathways were the most common in this study, especially in the PC group, where there were large metabolic differences when compared with the other two groups. The present study showed that nutrient metabolism in the intestinal flora of broilers with NE was generally downregulated, and the pathways related to intestinal flora metabolism were more enriched in the NC group, which was consistent with previous findings [43]. Bacteroidota are the main carbohydrate-degrading bacteria in the intestine that contribute to the degradation of complex pectins. Therefore, the enrichment of the carbohydrate metabolic pathway in the NC group may be due to the high abundance of Bacteroidota, which degrade complex polysaccharides and indigestible carbohydrates to generate short-chain fatty acids and maintain intestinal health [58]. Microbial catabolism of amino acids produces a large number of byproducts, including amines, phenols, indoles, and short-chain fatty acids [59], which can have beneficial or detrimental effects on epithelial cells, depending on the concentration of metabolic byproducts in the lumen. For example, aromatic amino acids are metabolized by colonies to produce tryptamine and indoles. Indoles play an important role in host defense by enhancing intestinal barrier function and downregulating the proinflammatory cytokines [60]. We speculated that the healthier gut in the NC group may be related to the products of carbohydrate and amino acid metabolism formed by the intestinal flora. Bacteroidota, *Propionibacterium*, *Fusobacterium*, *Lactobacillus*, and *Streptococcus* play important roles in the protein hydrolysis process [61], and the downregulation of amino acid metabolic pathways in the PC group of microorganisms may be due to a decline in Bacteroidota and *Lactobacillus* abundance. In the PC group, genes related to disease immunity, metabolic diseases, and energy metabolism were upregulated, which suggested that during the infection of chickens with *C. perfringens*, the intestinal flora competed with the host for energy, and more energy allocation was required for stimulation of the immune system, which may have resulted in the reduction in chicken growth performance. According to this study, the microbial communities in normal chickens were relatively healthier, and the ileal flora structure and function of the tannic acid groups were predicted to be similar to those of the NC group chickens. Therefore, we conclude that the addition of tannic acid could alleviate ileal microflora disorder in broiler chickens with NE. Taken together, the mechanism underlying the effect of tannic acid on intestinal health may involve changes in the intestinal microflora; however, relevant metabolomic analyses are still required to explore the effects of tannic acid on metabolites, which can further help us to establish the relationship among tannic acid, intestinal flora, metabolites, and intestinal health.

To investigate the correlation between intestinal flora and growth performance, immunity, and mRNA expression of intestinal barrier genes and nutrient transport carriers, simple correlation analysis was performed. Correlation analysis can indicate the correlation between indicators but cannot determine a causal relationship. The results suggested that the abundance of *Lactobacillus salivarius* was positively correlated with growth performance and significantly negatively correlated with MPO, CRP, ET, and zonulin, which may be because *Lactobacillus salivarius* can inhibit inflammation [62, 63] and improve intestinal barrier function [41]. The increase in the abundance of *Thermosinus carboxydivorans* and *Thermosinus* after the addition of tannic acid was significantly and positively correlated with the mRNA expression of *GLUT2*, which suggested that the regulation of intestinal flora by tannic acid may be related to nutrient translocation and absorption. However, the specific mechanisms require further investigation.

## Conclusions

The present study demonstrated that tannic acid supplementation is apparently effective in reducing NE in broiler chickens. The protective effects of dietary tannic acid supplementation against NE challenge were evidenced by the suppression of coccidia and *C. perfringens* colonization and invasion, regulation of intestinal flora, alleviation of inflammatory reaction, and improvement in intestinal barrier function and nutrient transport. As the amount of additive tannic acid increases, the body weight, mRNA expression of nutrient transport carriers, and villus height, V/C also increase in the form of a quadratic curve, while the intestinal barrier-related indices (zonulin and ET content in serum), coccidia oocyst counts, cecum *C. perfringens* concentration, and immune indices in serum decrease linearly. After comprehensive consideration of the results, we recommend tannic acid supplementation to broiler feed at a dose of 500–750 mg/kg.

## Data Availability

All data generated or analyzed during this study are available from the corresponding author upon request. Datasets supporting the conclusions of this study are included in this article. The 16S gene sequencing data can be obtained from the following website: https://www.ncbi.nlm.nih.gov/sra/PRJNA897828; the accession number is PRJNA897828.

## Abbreviations

BW: Body weight
CRP: C-reactive protein
ELISA: Enzyme-linked immunosorbent assay
ET: Endotoxin
F/G: The ratio of feed intake and body weight gain
GLUT2: Glucose transporter 2
I-FABP: Intestinal fatty acid binding protein
IgA: Immunoglobulin A
IgM: Immunoglobulin M
MPO: Myeloperoxidase
NE: Necrotic enteritis
PEPT1: Peptide-transporters 1
sIgA: Secretory immunoglobulin A
SGLT1: Sodium glucose cotransporter 1
V/C: The ratio of villus height to crypt depth
ZO-1: Zonula occludens-1
